# The Effects of Transcranial Direct Current Stimulation on Dual-Task Interference Depend on the Dual-Task Content

**DOI:** 10.3389/fnhum.2021.653713

**Published:** 2021-03-26

**Authors:** Takehide Kimura, Fuminari Kaneko, Takashi Nagamine

**Affiliations:** ^1^Graduate School of Health Sciences, Sapporo Medical University, Sapporo, Japan; ^2^Department of Physical Therapy, Faculty of Health Sciences, Tsukuba International University, Tsuchiura, Japan; ^3^First Division of Physical Therapy, School of Health Sciences, Sapporo Medical University, Sapporo, Japan; ^4^Department of Rehabilitation Medicine, Keio University School of Medicine, Tokyo, Japan; ^5^Department of Systems Neuroscience, School of Medicine, Sapporo Medical University, Sapporo, Japan

**Keywords:** dual-task, dual-task interference, dual-task content, transcranial direct continuous stimulation, dorsolateral prefrontal cortex

## Abstract

Recently, some studies revealed that transcranial direct current stimulation (tDCS) reduces dual-task interference. Since there are countless combinations of dual-tasks, it remains unclear whether stable effects by tDCS can be observed on dual-task interference. An aim of the present study was to investigate whether the effects of tDCS on dual-task interference change depend on the dual-task content. We adopted two combinations of dual-tasks, i.e., a word task while performing a tandem task (word-tandem dual-task) and a classic Stroop task while performing a tandem task (Stroop-tandem dual-task). We expected that the Stroop task would recruit the dorsolateral prefrontal cortex (DLPFC) and require involvement of executive function to greater extent than the word task. Subsequently, we hypothesized that anodal tDCS over the DLPFC would improve executive function and result in more effective reduction of dual-task interference in the Stroop-tandem dual-task than in the word-tandem dual-task. Anodal or cathodal tDCS was applied over the DLPFC or the supplementary motor area using a constant current of 2.0 mA for 20 min. According to our results, dual-task interference and the task performances of each task under the single-task condition were not changed after applying any settings of tDCS. However, anodal tDCS over the left DLPFC significantly improved the word task performance immediately after tDCS under the dual-task condition. Our findings suggested that the effect of anodal tDCS over the left DLPFC varies on the task performance under the dual-task condition was changed depending on the dual-task content.

## Introduction

When an individual performs two tasks simultaneously, the performance of either one or both tasks may often be impaired. This impairment of performance under a dual-task condition is defined as dual-task interference (Ebersbach et al., 1995). In our daily life, dual-task interference is known to cause various accidents, such as falls or traffic accidents (Lundin-Olsson et al., 1997; Chaparro et al., 2005). For example, when elderly individuals perform a cognitive task while walking, a reduction in gait speed and an increment in the cadence and stride duration has been observed (Gomes et al., 2016). Moreover, when young people talk on a cell phone (even when using hands-free cell phone) while driving, the driving performance has been shown to be impaired (Strayer and Drews, 2007). As such, dual-task interferences have the potential to cause unexpected accidents. Thus, exploring ways to reduce dual-task interference represents an efficient way to decrease the risk of accidents.

Recently, transcranial direct current stimulation (tDCS) came into focus for the reduction of dual-task interference. tDCS is a non-invasive neuromodulation technique and considered to modulate the cortical excitability of target brain regions by applying constant, weak electric current between two electrodes on the scalp (Nitsche et al., 2008). The stimulatory effects of tDCS are considered to differ depending on the current polarity under conventional stimulation parameters: anodal tDCS enhances cortical excitability, whereas cathodal tDCS inhibits cortical excitability in the brain region affected by the electrode (Nitsche and Paulus, 2000). tDCS is an easy-to-use and low-cost stimulation tool with few side effects (Nitsche et al., 2008). Taking advantage of this feature, for example, the usefulness of tDCS as a rehabilitation tool has been investigated for patients with stroke (Di Pino et al., 2014; Elsner et al., 2016). Thus, tDCS has potential in the clinical field as a new method to modulate brain excitability.

If we want to reduce dual-task interference using tDCS, the dorsolateral prefrontal cortex (DLPFC) may be one of the target brain regions. Neuroimaging studies have suggested that the DLPFC is associated with dual-task performances (D’Esposito et al., 1995; Koechlin et al., 1999), and that anodal tDCS over the left and right DLPFC reduces dual-task interference (Zhou et al., 2014, 2015; Strobach et al., 2015, 2018; Wrightson et al., 2015; Manor et al., 2016). Especially, focusing on the findings of the previous studies related to the accidents in our daily life, the anodal tDCS over the left DLPFC (intensity: 1.5–2.0 mA, duration: 15–20 min) reduced dual-task interference caused by the combination of a motor task (standing or walking) and a cognitive task (serial-subtraction task) (Zhou et al., 2014, 2015; Wrightson et al., 2015; Manor et al., 2016). However, as there are countless combinations of dual-tasks, tDCS may not be equally effective in reducing dual-task interferences for all dual-tasks. Moreover, the previous studies have used the serial-subtraction task as the cognitive task, and it was unclear whether differences in the contents of the cognitive task might have a dual-task influence on the effect of tDCS on dual-task interference. If this point can be clarified, we may be able to determine whether tDCS is effective in reducing dual-task interference, based on the dual-task content. Therefore, this study aimed to investigate whether the effects of tDCS on dual-task interference changed depending on the dual-task content.

In this study, we adopted a tandem task as the motor task. Previous studies have adopted the walking and standing task (Zhou et al., 2014, 2015; Wrightson et al., 2015; Manor et al., 2016) as the motor task; however, we are aware that a walking task includes several measurement parameters and may be performed using different strategies by the participant (e.g., decreasing the walking speed to improve balance, or increasing it at the expense of balance). Additionally, the present study recruited healthy young people, and a standing task might have been too easy for them. Therefore, we tried to avoid the ceiling effect by using the tandem task, which is more difficult than the standing task.

In the present study, we adopted a word task and a Stroop task as cognitive tasks. Subsequently, dual-tasks were constructed by combining the word task and the tandem task (word-tandem dual-task) or the Stroop task and the tandem task (Stroop-tandem dual-task). The word task required to read out loud the name of the color displayed in black. The Stroop task was a classical Stroop task and required to read out loud the name of the color that was used to spell the displayed word. We adopted these cognitive tasks because we attempted to create a difference in the cognitive functions and brain regions that would be involved in the tasks. Previous tDCS studies have shown that anodal tDCS over the left DLPFC reduced dual-task interference caused by the combination of a motor task (standing or walking) and a cognitive task (serial-subtraction task) (Zhou et al., 2014, 2015; Wrightson et al., 2015; Manor et al., 2016). Focusing on the cognitive task, the serial-subtraction task was expected to enhance DLPFC excitability (Vansteensel et al., 2014) and require the involvement of executive function (Strobach and Antonenko, 2016). In addition, other tDCS studies have shown that anodal tDCS over the left DLPFC improved executive function (Strobach and Antonenko, 2016; Imburgio and Orr, 2018). Given that anodal tDCS over the DLPFC improved executive function and resulted in improved task performance related to executive function under the single-task condition, we hypothesized that anodal tDCS over the DLPFC would also improve a task performance related to executive function, even under the dual-task condition. As a result of this improvement in task performance under the dual-task condition, the other task included in the dual-task could be performed more easily, and dual-task interference might be reduced. To clarify this hypothesis, we adopted the Stroop task and the word task. The Stroop task required executive function (Strobach and Antonenko, 2016). In addition, the Stroop task enhanced more brain regions (e.g., DLPFC) than the word task (Adleman et al., 2002). Because the DLPFC is was one of important brain regions related to the executive function (Miller and Cohen, 2001), we expected that the Stroop task would require greater executive function resources than the word task. As a result, we predicted that the effect of anodal tDCS over the left DLPFC would lead to greater improvement in dual-task performance when the Stroop task (i.e., the Stroop-tandem dual-task) would be included than when it would not be included (i.e., the word-tandem dual-task).

## Materials and Methods

### Subjects

Ten healthy males (age 22.8 ± 1.6 years; height 172.1 ± 5.1 cm; weight 66.2 ± 7.0 kg; mean ± SD) participated in the present study. We conducted a sample size calculation using the open source, web-based application ‘‘Power ANalysis for GEneral Anova designs’’ (PANGEA [ver.3.9])^1^ (Westfall, 2016). We calculated the sample size required for a three-way repeated-measures analysis of variance (ANOVA) with tDCS placement [DLPFC vs. supplementary motor area (SMA)], tDCS polarity (anodal vs. cathodal), and time (pre, post 0, post 20, and post 40) as the within-subject factors. In the present study, we also conducted a four-way repeated-measures ANOVA. However, because the number of subjects was underestimated when the sample size was calculated with the four-way repeated-measures ANOVA, we calculated the sample size with the three-way repeated-measures ANOVA. The calculation was carried out with the number of replicates at 3, and the effect size at Cohen’s *d* = 0.45. This Cohen’s *d* value is considered the effect size of the medium (Cohen, 1988). Because there were no comparable studies before the present study, we adopted this medium effect size which was commonly used and was a default value of PANGEA. The power analysis result showed that at least nine participants were needed to produce sufficient statistical power (i.e., power > 0.80). Subsequently, we recruited 10 participants, accounting for the possibility of participant dropout. All subjects were native Japanese speakers and were right-handed as assessed using the Edinburgh Handedness Inventory (Oldfield, 1971). We confirmed that they had normal or corrected-to-normal vision and could read a list of words displayed on a computer screen that was placed 2 m in front of a chair they sat on. Subjects were excluded from the present study if they met any of the following criteria: (1) history of head injury or head surgery, (2) history of neurological, otological (e.g., dizziness), ophthalmological (e.g., color blindness), or cardiovascular illness, (3) history of orthopedic illness of the lower limb, (4) metallic implants, and (5) problems to communicate. All subjects signed an informed consent form. The present study was performed in accordance with the Declaration of Helsinki (last modified in 2013). The protocol of the present study was approved by the Ethics Committee of Sapporo Medical University.

### tDCS Settings

Transcranial direct current stimulation was delivered by a battery-driven constant current stimulator (DC-STIMULATOR, neuroConn GmbH, Ilmenau, Germany) via a saline-soaked pair of surface sponge electrodes (7 cm × 5 cm). To apply tDCS over the left DLPFC, one electrode was placed over the F3 according to the International 10–20 System for Electroencephalography Electrode Placement, and the other electrode (reference electrode) was placed over the right supraorbital region. To apply tDCS to the SMA, one electrode was placed over the Cz, and the other electrode (reference electrode) was placed over the right supraorbital region. We applied tDCS over the SMA as a control placement for the DLPFC. The stimulation protocol was a constant current of 2.0 mA applied for 20 min with fade-in/fade-out periods of 7 s. During the tDCS application, the subject was seated in a chair.

### Content of Single-Task

#### Tandem Task

The subject stood on the force platform (9285, Kistler Japan, Tokyo, Japan) in the tandem Romberg posture with bare feet and open eyes. The subject was instructed to place the right foot behind the left foot so that the left heel was in contact with the right toe. The relaxed arms were beside the body. The subject was instructed to distribute the body weight equally between the left and right leg and to stand as motionless as possible while gazing at a point at eye level at a distance of 2 m. This point was displayed on a 24-inch computer screen. To analyze a postural sway, the output form the force platform was introduced to the computer through an analog-to-digital converter (PH-790, DKH Corp., Tokyo, Japan) at a sampling frequency of 1,000 Hz. After that, we calculated the total path length of the center of pressure (COP path length) (cm) by using TRIAS software (ver.3.9, DKH Corp., Tokyo, Japan). The COP path length is the most common method of measuring the postural sway, and is used to detect dual-task interference in the previous studies (Shumway-Cook et al., 1997; Melzer et al., 2001). At the beginning of the tandem task, the subject tried to hold the center of pressure within 4 cm^2^ rectangle for 5 s. If the subject could hold it for 5 s, we started measuring the COP path length for 30 s and defined this result of COP path length for 30 s as the tandem task performance.

#### Word Task, and Stroop Task

The subject sat on a chair and fixated the center of a 24-inch computer screen that was placed 2 m in front of it. The subject was required to read out loud as quickly as possible a list of words displayed on a computer screen for 30 s. In the word task, the list of 42 words consisted of the four displayed words “red,” “blue,” “green,” and “yellow.” The 42 words (7 rows × 6 columns) were randomly listed and displayed in black. The stimulus covered approximately between 10.4° and 14.0° of the visual angles. The Stroop task employed the same list of words used in the word task but each word was not displayed in black but one of these four colors instead. The subject was required to read out loud the color name that was incongruent with the displayed word (e.g., the subject was required to say “red” when the word “blue,” “green,” or “yellow” was displayed in red). We defined the number of read out (words) within 30 s as the word task performance, and defined the concordant word number (words) within 30 s as the Stroop task performance. In the Stroop task, in particular, we observed that there were two types of subjects in the pilot study: those with a fast reaction-time but several errors, and those with a slow reaction-time but few errors (i.e., speed-accuracy trade-off). Thus, in the present study, we allowed the subjects to rephrase if they gave a spur-of-the-moment incorrect answer. As a result, the number of errors in both the word task and the Stroop task were zero for all the subjects. Through these procedures, we expected that the concordant word-number would reflect the two factors; i.e., the reaction-time and the number of errors.

### Content of Dual-Task

#### Word-Tandem Dual-Task, and Stroop-Tandem Dual-Task

**FIGURE 1:**
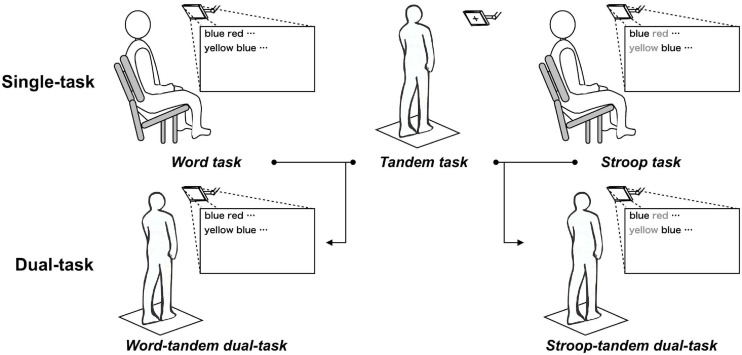
Construction of the word-tandem task. Single-tasks consisted of the word, Stroop, or tandem task. Dual-tasks were constructed from the word task and tandem task (word-tandem dual-task) or from the Stroop task and tandem task (Stroop-tandem dual-task).

The dual-task included performing the word task while performing the tandem task (word-tandem task) and the Stroop task while performing the tandem task (Stroop-tandem task). An overview is shown in Figure 1. The subject stood in the tandem Romberg posture with bare feet on the force platform and was instructed to focused on the display. After the preparation to perform the tandem task (i.e., the subject was able to hold the center of pressure within 4 cm^2^ rectangle for 5 s), the subject signaled this to the investigator by saying “yes,” and the word list of word or the Stroop task appeared 5 s after this acoustic cue. We measured each task performance under the dual-task for 30 s.

### Experimental Procedures

**FIGURE 2:**
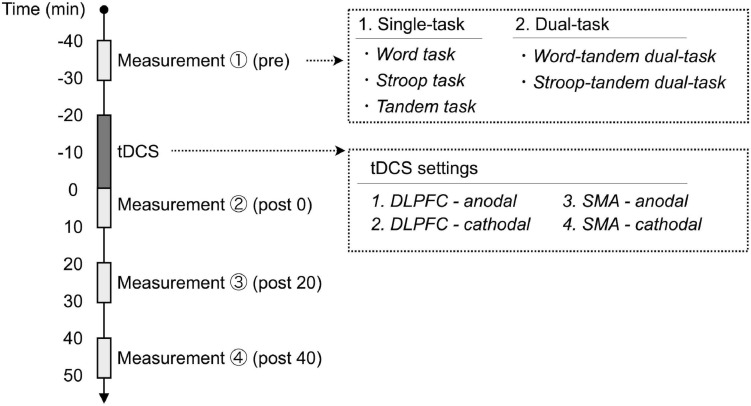
Experimental procedures. Each subject participated in four experimental sessions with different stimulation parameters of transcranial direct current stimulation (tDCS). We measured single- and dual-task performances before and after applying tDCS. Each task performance was assessed before applying tDCS (pre), immediately after applying tDCS (post 0), 20 min after applying tDCS (post 20), and 40 min after applying tDCS (post 40). DLPFC, dorsolateral prefrontal cortex; SMA, supplementary motor area.

The paradigm is illustrated in Figure 2. Each subject participated in four experimental sessions composed of combination of two different tDCS stimulation polarities and two stimulation sites including the following: (1) anodal tDCS over the DLPFC, (2) cathodal tDCS over the DLPFC, (3) anodal tDCS over the SMA, and (4) cathodal tDCS over the SMA. Subjects, but not experimenters, were blinded to these tDCS settings (single-blind). All subjects underwent a randomized crossover in the four experimental sessions separated by at least 6 days. Before applying tDCS (pre), immediately after applying tDCS (post 0), 20 min after applying tDCS (post 20), and 40 min after applying tDCS (post 40), the subjects completed the single-tasks (word task, Stroop task, and tandem task) and dual-tasks (word-tandem dual-task and Stroop-tandem dual-task) three times in each time point. The order of performing these tasks were random (e.g., word task, Stroop-tandem dual-task, tandem task…), and the subjects rested for 30 s between performing each task.

At the beginning of each experimental session, the subjects participated in a practice session to familiarize themselves with the single-tasks and the dual-tasks, respectively. Taking the tandem task under the single-task condition as an example, at first, subjects performed the tandem task for 15 s and performed it 16 times. Resting of 15 s was allowed between performing tandem tasks. In this way, to prevent the experiment from becoming too long and the subject from becoming fatigued, we halved the duration of performing each task and resting in the practice session compared to the experimental session. We calculated a difference between the 15th and 16th tandem task performance. If this difference was less than 10% of the 15th tandem task performance, we judged that the subject was familiar with the tandem task. In other cases, subjects performed an additional practice 8 times, and re-judged as a same way using the last and next to last task performance (e.g., 23rd and 24th tandem task performance). This definition was based on previous studies (Boggio et al., 2006). Additionally, we confirmed that the practice session did not cause fatigue in the subjects and did not decrease their performances in subsequent tasks in the pilot study.

### Statistics

The statistical analysis was performed using SPSS (version 22; IBM, Corp., Armonk, NY, United States). At first, we applied the Shapiro–Wilk test to examine the normality and equality of variance of the variables. The results of the task performances under the single- and dual-task condition were entered in a three-way repeated-measures ANOVA with tDCS placement (DLPFC vs. SMA), tDCS polarity (anodal vs. cathodal), and time (pre, post 0, post 20, vs. post 40) as the with-in factors. In addition, to assess the effect of tDCS on the changes in dual-task interference, we performed combined analysis of the single-task and dual-task. That is, we applied a four-way repeated-measures ANOVA with condition (single-task vs. dual-task), tDCS placement (DLPFC vs. SMA), tDCS polarity (anodal vs. cathodal), and time (pre, post 0, post 20, vs. post 40) as the within-subject factors. This combined analysis was conducted on the following results: (1) tandem task performance [under the single-task and word-tandem dual-task conditions], (2) tandem task performance [under the single-task and Stroop-tandem dual-task conditions], (3) word task performance [under the single-task and word-tandem dual-task conditions], (4) Stroop task performance [under the single-task and Stroop-tandem dual-task conditions]. When Mauchly’s test was significant (i.e., we confirmed that the sphericity assumptions were violated), results with Greenhouse–Geisser-adjusted degrees of freedom were reported. When a significant interaction was detected, Bonferroni’s or Dunnett’s *post hoc* analysis was performed. To evaluate the difference of task difficulty between the word task and the Stroop task and to confirm a Stroop interference, we calculated the average value of the number of read out (i.e., word task performance) and the concordant word number (i.e., Stroop task performance) before applying tDCS (i.e., pre) in each participant for all tDCS settings. Afterward, the difference in task performance between the word and the Stroop task was compared using the paired *t*-test. The statistical significance threshold was set as *p* < 0.05. Additionally, we performed a *post hoc* power analysis of this study using PANGEA (ver.3.9)^2^ that evaluated ANOVA design, and we obtained power (1-β) (Westfall, 2016).

## Results

All subjects completed the entire experiment, and no tDCS side effects were observed during and after the experiment. The concordant word number (i.e., the Stroop task performance) was significantly lower than the number of read out (i.e., the word task performance) before applying tDCS [*t*(9) = 8.894, *p* < 0.001, the average of concordant word number: 59.2 ± 8.5 words, the average of number of read out: 83.6 ± 9.4 words]. In addition, we calculated the approximate reaction times by dividing 30 s by the concordant word number (i.e., the Stroop task performance) or the number of read outs (i.e., the word task performance). As a result, the approximate reaction time of the Stroop task was 518.2 ± 85.3 ms, and the approximate reaction time of the word task was 363.6 ± 42.2 ms. The results of the task performance under the single-task condition and under the dual-task condition are shown in Tables 1–3, respectively. All statistical analysis results are presented in Supplementary Tables 1–6.

**TABLE 1:** The result of the COP path length (cm) in the tandem task under the single- and dual-task condition.

	Single-task				Dual-task (word-tandem dual-task)				Dual-task (stroop-tandem dual-task)			
	DLPFC		SMA		DLPFC		SMA		DLPFC		SMA	
	Anodal	Cathodal	Anodal	Cathodal	Anodal	Cathodal	Anodal	Cathodal	Anodal	Cathodal	Anodal	Cathodal
Pre	106.0 (21.4)	94.7 (16.7)	98.3 (19.1)	106.7 (16.3)	110.2 (23.6)	108.4 (21.6)	110.1 (26.9)	117.7 (27.0)	112.7 (27.0)	112.0 (20.6)	114.2 (28.6)	117.3 (25.2)
Post 0	103.0 (21.8)	95.5 (14.6)	99.4 (22.2)	105.1 (18.4)	112.1 (28.5)	109.9 (21.6)	110.4 (24.3)	119.7 (28.4)	109.6 (26.3)	111.6 (21.7)	107.2 (23.4)	117.4 (27.6)
Post 20	101.3 (19.0)	97.0 (17.2)	98.4 (25.1)	103.3 (18.2)	109.1 (27.8)	107.1 (21.4)	108.7 (23.4)	117.5 (25.1)	108.1 (23.7)	110.8 (23.7)	110.0 (24.9)	114.2 (22.5)
Post 40	97.7 (17.2)	99.0 (17.8)	98.4 (21.1)	104.0 (19.1)	106.2 (21.9)	108.6 (20.5)	111.9 (24.3)	112.0 (20.4)	108.2 (22.1)	109.5 (17.7)	115.7 (25.9)	114.9 (24.5)

*COP, center of pressure; DLPFC, dorsolateral prefrontal cortex; SMA, supplementary motor area. Values are mean (standard deviation).*

**TABLE 2:** The result of the number of read out (words) in the word task under the single- and dual-task condition.

	Single-task				Dual-task (word-tandem dual-task)			
	DLPFC		SMA		DLPFC		SMA	
	Anodal	Cathodal	Anodal	Cathodal	Anodal	Cathodal	Anodal	Cathodal
Pre	83.4 (9.3)	84.0 (9.5)	84.9 (10.0)	81.9 (9.9)	82.8 (11.0)	84.1 (8.1)	85.7 (10.3)	81.7 (9.7)
Post 0	85.8 (8.9)	85.8 (8.5)	85.7 (9.7)	81.9 (8.8)	86.9* (10.0)	85.8 (8.0)	85.4 (10.0)	83.2 (9.4)
Post 20	84.3 (10.6)	85.0 (8.9)	84.2 (9.6)	83.0 (9.8)	84.2 (11.4)	85.9 (9.1)	84.1 (10.6)	82.9 (9.4)
Post 40	83.7 (11.3)	84.0 (9.7)	84.3 (9.6)	82.3 (9.8)	84.2 (10.7)	83.4 (9.3)	83.8 (9.8)	82.8 (8.1)

DLPFC, dorsolateral prefrontal cortex; SMA, supplementary motor area. Values are mean (standard deviation). *Significant difference compared with Pre time point, at p < 0.05.

**TABLE 3:** The result of the concordant word number (words) in the Stroop task under the single- and dual-task condition.

	Single-task				Dual-task (stroop-tandem dual-task)			
	DLPFC		SMA		DLPFC		SMA	
	Anodal	Cathodal	Anodal	Cathodal	Anodal	Cathodal	Anodal	Cathodal
Pre	57.4 (9.1)	61.6 (8.1)	60.5 (7.5)	57.5 (9.7)	56.8 (10.2)	61.3 (7.6)	59.4 (9.8)	56.5 (8.3)
Post 0	57.0 (10.7)	61.3 (8.0)	60.0 (8.7)	56.4 (7.3)	58.2 (10.6)	62.5 (8.7)	59.0 (8.2)	56.4 (7.6)
Post 20	56.9 (8.6)	60.6 (7.9)	58.5 (8.86	56.9 (7.9)	57.5 (10.7)	61.2 (7.8)	58.3 (8.2)	57.1 (7.6)
Post 40	56.2 (11.0)	60.9 (9.9)	59.9 (9.0)	55.9 (10.1)	58.2 (10.6)	60.7 (9.2)	58.6 (9.7)	57.2 (9.1)

*DLPFC, dorsolateral prefrontal cortex; SMA, supplementary motor area. Values are mean (standard deviation).*

### Tandem Task (Table 1)

#### The Result of the COP Path Length Under the Single-Task Condition

There was no significant interaction: placement × polarity × time [*F*(3,27) = 2.850, *p* = 0.056, $\eta_{\text{p}}^{2}$ = 0.024, 1-β = 0.165], but, was a significant interaction: placement × polarity [*F*(1,9) = 6.285, *p* = 0.033, $\eta_{\text{p}}^{2}$ = 0.411, 1-β = 1.000]. In response to the significant interaction of placement × polarity, we analyzed a simple main effect. As a result, there was a significant difference between DLPFC and SMA in the cathodal polarity (*p* = 0.006). No other simple main effects were found (difference between DLPFC and SMA in the anodal polarity: *p* = 0.399, difference between anodal and cathodal polarity in the placement of DLPFC: *p* = 0.102, difference between anodal and cathodal polarity in the placement of SMA: *p* = 0.143).

#### The Results of the COP Path Length Under the Dual-Task Conditions

Under the word-tandem dual-task condition, there was a significant interaction: placement × polarity × time [*F*(3,27) = 3.026, *p* = 0.047, $\eta_{\text{p}}^{2}$ = 0.251, 1-β = 0.950]. However, no other main effects or interactions (placement × time, placement × polarity, and polarity × time) were found. Under the Stroop-tandem dual-task condition, there were no significant interactions and main effects.

#### The Results of the Combined Analysis Under the Single-Task and Word-Tandem Dual-Task Conditions

There was a significant interaction of placement × polarity × time [*F*(3,27) = 3.694, *p* = 0.024, $\eta_{\text{p}}^{2}$ = 0.291, 1-β = 1.000]. Given the significant interaction of placement × polarity × time, we analyzed the simple interaction effect. As a result, there was a significant interaction of placement × polarity in the pre, post 0, and post 20 time points {pre: [*F*(1,19) = 9.966, *p* = 0.005], post 0: [*F*(1,19) = 14.753, *p* = 0.001], and post 20: [*F*(1,19) = 7.333, *p* = 0.014]}. Summaries of *post hoc* comparisons were as follows: (1) the COP path length was significantly longer by applying anodal tDCS over the DLPFC than by applying cathodal tDCS over the DLPFC in the pre (*p* = 0.038) and post 0 (*p* = 0.040) time points, (2) the COP path length was significantly longer by applying cathodal tDCS over the SMA than by applying anodal tDCS over the SMA in the pre (*p* = 0.028) and post 0 (*p* = 0.008) time points, (3) the COP path length was significantly longer by applying cathodal tDCS over the SMA than by cathodal tDCS over the DLPFC in the pre (*p* < 0.001), post 0 (*p* < 0.001), and post 20 (*p* = 0.014) time points.

#### The Results of Combined Analysis Under the Single-Task and Stroop-Tandem Dual-Task Conditions

There was a significant main effect of condition [*F*(1,9) = 15.930, *p* = 0.003, $\eta_{\text{p}}^{2}$ = 0.639, 1-β = 1.000], and the COP path length was significantly longer under the Stroop-tandem dual-task condition than under the single-task condition (*p* = 0.003). Other significant main effects and interactions were not observed.

### Word Task (Table 2)

#### The Result of the Number of Read Out Under the Single-Task Condition

There were no significant interactions and main effects.

#### The Result of the Number of Read Out Under the Dual-Task Condition

There was a significant interaction: placement × polarity × time [*F*(3,27) = 4.498, *p* = 0.011, $\eta_{\text{p}}^{2}$ = 0.333, 1-β = 1.000]. In response to the significant interaction of placement × polarity × time, we analyzed a simple interaction effect. As a result, there was a significant interaction placement × time in the anodal polarity [*F*(3,27) = 4.019, *p* = 0.017]. *Post hoc* comparisons (Dunnett’s test) revealed that the number of read out in the post 0 time point was significantly more improved than in the pre time point by applying anodal tDCS over the DLPFC (*p* < 0.001). On the other hand, there was no significant interaction (placement × time) [*F*(3,27) = 1.406, *p* = 0.263] and main effects [placement: *F*(1,9) = 1.838, *p* = 0.308, time: *F*(3,27) = 2.585, *p* = 0.074] in the cathodal polarity.

#### The Results of Combined Analysis Under the Single-Task and Word-Tandem Dual-Task Conditions

There was a significant main effect of time [*F*(3,27) = 4.793, *p* = 0.008, $\eta_{\text{p}}^{2}$ = 0.348, 1-β = 1.000]. *Post hoc* comparisons revealed that the number of read outs in the post 0 time point was significantly more improved than that in the pre time point (*p* = 0.011). Other significant main effects and interactions were not observed.

### Stroop Task (Table 3)

#### The Result of the Concordant Word Number Under the Single-Task Condition

There was no significant interaction: placement × polarity × time [*F*(3,27) = 1.246, *p* = 0.313, $\eta_{\text{p}}^{2}$ = 0.122, 1-β = 0.641], but, was a significant interaction: placement × polarity [*F*(1,9) = 7.363, *p* = 0.024, $\eta_{\text{p}}^{2}$ = 0.450, 1-β = 1.000]. In response to the significant interaction of placement × polarity, we analyzed a simple main effect. As a result, there was a significant difference between DLPFC and SMA in the anodal and cathodal polarity (*p* = 0.013, *p* < 0.001), and a significant difference between anodal and cathodal polarity in the DLPFC and SMA placement (*p* < 0.001, *p* = 0.017).

#### The Result of the Concordant Word Number Under the Dual-Task Condition

There were no significant interactions and main effects.

#### The Results of Combined Analysis Under the Single-Task and Stroop-Tandem Dual-Task Conditions

There were significant interactions of placement × polarity [*F*(1,9) = 5.779, *p* = 0.040, $\eta_{\text{p}}^{2}$ = 0.391, 1-β = 1.000] and condition × placement [*F*(1,9) = 6.749, *p* = 0.029, $\eta_{\text{p}}^{2}$ = 0.429, 1-β = 1.000]. Given these significant interactions, we analyzed the simple main effect. As a result, there was a significant difference between the DLPFC and SMA in anodal and cathodal polarity (*p* = 0.017 and *p* < 0.001, respectively) and a significant difference between anodal and cathodal polarity in DLPFC and SMA placement (*p* < 0.001 and *p* = 0.002, respectively). In addition, there was a significant difference between the DLPFC and SMA under the dual-task condition (*p* = 0.018).

## Discussion

In the present study, we investigated whether anodal/cathodal tDCS over the left DLPFC/SMA would impact dual-task interference depending on the dual-task content. As a result of the combined analysis, there was no significant interaction including the condition (single-task vs. dual-task) as a factor. This result indicated that both single- and dual-task performance were not affected by other factors, such as tDCS placement (DLPFC vs. SMA), tDCS polarity (anodal vs. cathodal), and time (pre, post 0, post 20, vs. post 40). That is, the results of the combined analysis suggested that no tDCS setting specifically changed dual-task performance or reduced dual-task interference. Conversely, as results of analysis of each task performance under the single- or dual-task condition, anodal tDCS over the left DLPFC significantly improved word task performance under the dual-task condition. Taking into account the results of the combined analysis, we considered that anodal tDCS over the left DLPFC improved the word task performance under the single- and dual-task performance, but the degree of this improvement was greater under the dual-task condition than under the single-task condition. Cathodal tDCS over the left DLPFC and anodal/cathodal tDCS over the SMA did not affect task performance.

Previous studies have shown that anodal tDCS over the left DLPFC reduced the dual-task interference constituting of motor task and cognitive task in the younger and older adults (Zhou et al., 2014, 2015; Wrightson et al., 2015; Manor et al., 2016). In contrast, in this study, no tDCS setting affected the dual-task interference. We speculated that this difference in the results of our study and the previous studies may have been due to the content of the cognitive task constituted of the dual-task. Previous studies have used the serial-subtraction task as the cognitive task (Zhou et al., 2014, 2015; Wrightson et al., 2015; Manor et al., 2016), which requires the “updating” function (Ogden et al., 2014; Bristow et al., 2016); one of the brain’s executive function. The executive function is considered to be composed of three executive core components: “updating (constant monitoring and tracking of working memory representations)”, “shifting (switching between tasks or mental sets)”, and “inhibition (to deliberately inhibit dominant, automatic, or prepotent responses)” (Miyake et al., 2000). For example, as information related to our study and the aforementioned studies, “updating,” “shifting,” and “inhibition” functions were involved in performing “the serial-subtraction task and word task,” “the dual-task,” and “the Stroop task,” respectively (Strobach and Antonenko, 2016). Importantly, these functions were considered to share common underlying cognitive processes to some extent; especially, “shifting” was considered to share more cognitive processes with “inhibition” than with “updating” (Friedman et al., 2006; Snyder et al., 2015). Focusing on the Stroop-tandem dual-task, the subjects in the present study performed the dual-task and the Stroop task as cognitive tasks related to the executive function. These tasks required the “shifting” and “inhibition” function, and these functions might share more cognitive processes, compared with the combination of “shifting” and “updating” functions adopted in the previous studies (Zhou et al., 2014, 2015; Wrightson et al., 2015; Manor et al., 2016) (i.e., the subjects in the aforementioned studies performed the dual-task and the serial-subtraction task). Additionally, several studies have shown that anodal tDCS over the left DLPFC improved the executive function (Strobach and Antonenko, 2016; Imburgio and Orr, 2018). Thus, we speculated that the improvement of the executive function induced by the tDCS was more effective in the combination of tasks in the aforementioned studies (Zhou et al., 2014, 2015; Wrightson et al., 2015; Manor et al., 2016). If the difference in the effect of tDCS between the present study and the aforementioned studies is due to the difference in the cognitive processes, it may indicate that the improvement in DLPFC excitability is more effective for the dual-tasks which require “shifting” and “updating” functions. Similarly, the result of the word task performance under the dual-task condition improved significantly immediately after applying tDCS. The word task was one of verbal fluency, and may require the “updating” function (Shao et al., 2014). Thus, the effect of anodal tDCS over the DLPFC might be more likely to occur with a combination of “updating (related to performing word task)” and “shifting (related to performing dual-task)” function, as indicated in the previous studies (Zhou et al., 2014, 2015; Wrightson et al., 2015; Manor et al., 2016).

Each task performance under the single-task condition remained unchanged after applying the anodal tDCS. A previous study has shown that anodal tDCS over the left DLPFC improved the Stroop task performance under the single-task condition (Angius et al., 2019). At first glance, the result of the aforementioned study and our study appears contradictory. This may be because there were some methodological differences between the two studies, such as the tDCS settings (e.g., size of surface sponge electrodes and stimulation time) or the Stroop task method (e.g., how to respond to the stimuli, number of colors used, etc.). Based on these differences of experimental protocol under the single-task condition, we considered that it may be difficult to conclude that anodal tDCS over the left DLPFC affected the Stroop task performance in the present study.

Cathodal tDCS did not affect any results in the present study. This might have been due to the difficulties in obtaining stable stimulation effects with cathodal tDCS. For example, several previous studies have reported that anodal tDCS over the M1 enhances the excitability of the M1, which was evaluated by measuring the amplitude of motor-evoked potentials by using transcranial magnetic stimulation (TMS) (Nitsche and Paulus, 2000, 2001; Liebetanz et al., 2002). In contrast, when cathodal tDCS is applied over the M1, the amplitude of motor-evoked potentials may be decreased (Nitsche and Paulus, 2000), unchanged (Strube et al., 2016), or increased (Batsikadze et al., 2013). Further, it has been reported that cathodal tDCS over the DLPFC does not change the cognitive task performance, despite anodal tDCS over the DLPFC improving the same task performance (Jacobson et al., 2012). As demonstrated in the previous studies, the stimulatory effect of cathodal tDCS over the M1 is unstable, and that over the DLPFC may not be obtained.

There are several limitations to our study. First, this study used only two types of dual-tasks. Whether our findings are applicable to all dual-tasks remains unknown. Second, our study had a limited sample size, and future studies should recruit large samples. Third, all subjects underwent a randomized crossover in the four experimental sessions. However, there may have been statistical differences between each session, especially in the tandem task (e.g., the difference in the COP path length under the single-task condition between the DLPFC and SMA in the cathodal polarity). If we adopted tasks with less variation in task performances between days, trials, and subjects, we might be able to evaluate the effect of tDCS in more detail. Fourth, we defined the concordant word number as the Stroop task performance, and the number of read outs as the word task performance. However, the reaction time is more commonly used to evaluate these task performances. The results regarding the reaction time would be easier to compare with the findings of other studies than the concordant word number or number of read outs.

## Conclusion

We expected that cathodal tDCS may not produce a sufficient change in task performance in this study; however, the effects of anodal tDCS over the DLPFC possibly indicated an improvement in word task performance under the dual-task condition, despite not having any affect on dual-task interferences. The important novel point of the present study was that it indicated that in dual-task performance, the effect of anodal tDCS over the DLPFC changes depending on the dual-task content. A future study may be needed to explore the key factors that influence the effect of tDCS on the dual-task interference.

## Data Availability Statement

The raw data supporting the conclusions of this article will be made available by the authors, without undue reservation, to any qualified researcher.

## Ethics Statement

The studies involving human participants were reviewed and approved by Ethics Committee of Sapporo Medical University. The patients/participants provided their written informed consent to participate in this study.

## Author Contributions

TK, FK, and TN contributed to the study conception and design. Material preparation, data collection and analysis were performed by TK and FK. The first draft of the manuscript was written by TK and FK. TN commented on previous versions of the manuscript. All authors approved the final version of the manuscript, and agreed to be accountable for all aspects of the work in ensuring that questions related to the accuracy or integrity of any part of the work are appropriately investigated and resolved.

## Conflict of Interest

The authors declare that the research was conducted in the absence of any commercial or financial relationships that could be construed as a potential conflict of interest.

## Footnotes

1 https://jakewestfall.shinyapps.io/pangea/

2 https://jakewestfall.shinyapps.io/pangea/
