# Supplementary material for: The Effects of Transcranial Direct Current Stimulation on Dual-Task Interference Depend on the Dual-Task Content
Source: Front Hum Neurosci. 2021 Mar 26;15:653713. doi: 10.3389/fnhum.2021.653713 (PMC8032873; doi:10.3389/fnhum.2021.653713)
Supplement: Supplementary file 1 [file Table_1.DOCX]

**Supplemental table S1.** Result of the three-way repeated-measures ANOVA on variable in the tandem task under the single- and dual-task condition

| **Single-task condition** | Tandem task | | | |  |  | |  |  |
| --- | --- | --- | --- | --- | --- | --- | --- | --- | --- |
|  | F value | p value | partial η^2^ | 1-β |  |  |  |  |  |
| Placement | 1.276 | 0.288 | 0.124 | 0.827 |  |  |  |  |  |
| Polarity | 0.018 | 0.896 | 0.002 | 0.064 |  |  |  |  |  |
| Time | 0.364 | 0.780 | 0.039 | 0.365 |  |  |  |  |  |
| Placement × Polarity | 6.285 | 0.033 | 0.411 | 1.000 |  |  |  |  |  |
| Placement × Time | 0.155 | 0.926 | 0.017 | 0.236 |  |  |  |  |  |
| Polarity × Time | 0.814 | 0.497 | 0.084 | 0.813 |  |  |  |  |  |
| Placement × Polarity × Time | 2.850 | 0.056 | 0.024 | 0.165 |  |  |  |  |  |
|  |  | |  |  |  |  | |  |  |
| **Dual-task condition** | Word-tandem dual-task | | | |  | Stroop-tandem dual-task | | | |
|  | F value | p value | partial η^2^ | 1-β |  | F value | p value | partial η^2^ | 1-β |
| Placement | 2.261 | 0.167 | 0.201 | 0.971 |  | 2.263 | 0.167 | 0.201 | 0.971 |
| Polarity | 1.724 | 0.221 | 0.161 | 0.922 |  | 1.358 | 0.274 | 0.131 | 0.851 |
| Time | 1.316 | 0.290 | 0.128 | 0.866 |  | 1.447 | 0.251 | 0.138 | 0.893 |
| Placement × Polarity | 4.123 | 0.073 | 0.314 | 1.000 |  | 0.455 | 0.517 | 0.048 | 0.549 |
| Placement × Time | 0.025 | 0.995 | 0.003 | 0.081 |  | 0.643 | 0.594 | 0.067 | 0.713 |
| Polarity × Time | 0.396 | 0.757 | 0.042 | 0.505 |  | 0.152 | 0.231 | 0.145 | 0.972 |
| Placement × Polarity × Time | 3.026 | 0.047 | 0.251 | 0.950 |  | 0.852 | 0.478 | 0.087 | 0.484 |

Abbreviations: ANOVA, analysis of variance
