# Supplementary material for: The Effects of Transcranial Direct Current Stimulation on Dual-Task Interference Depend on the Dual-Task Content
Source: Front Hum Neurosci. 2021 Mar 26;15:653713. doi: 10.3389/fnhum.2021.653713 (PMC8032873; doi:10.3389/fnhum.2021.653713)
Supplement: Supplementary file 2 [file Table_2.DOCX]

**Supplemental table S2.** Result of the three-way repeated-measures ANOVA on variable in the word task under the single- and dual-task condition

| **Single-task condition** | Word task | | | |
| --- | --- | --- | --- | --- |
|  | F value | p value | partial η^2^ | 1-β |
| Placement | 0.836 | 0.384 | 0.085 | 0.656 |
| Polarity | 1.883 | 0.203 | 0.173 | 0.941 |
| Time | 1.987 | 0.140 | 0.180 | 0.963 |
| Placement × Polarity | 2.505 | 0.148 | 0.218 | 0.998 |
| Placement × Time | 2.462 | 0.084 | 0.215 | 0.998 |
| Polarity × Time | 0.994 | 0.411 | 0.099 | 0.877 |
| Placement × Polarity × Time | 0.439 | 0.727 | 0.046 | 0.277 |
|  |  | |  |  |
| **Dual-task condition** | Word-tandem dual-task | | | |
|  | F value | p value | partial η^2^ | 1-β |
| Placement | 0.832 | 0.385 | 0.084 | 0.650 |
| Polarity | 0.595 | 0.460 | 0.061 | 0.507 |
| Time | 6.223 | 0.002 | 0.409 | 1.000 |
| Placement × Polarity | 1.762 | 0.217 | 0.172 | 0.987 |
| Placement × Time | 1.874 | 0.158 | 0.163 | 0.986 |
| Polarity × Time | 1.314 | 0.290 | 0.127 | 0.948 |
| Placement × Polarity × Time | 4.498 | 0.011 | 0.333 | 1.000 |

Abbreviations: ANOVA, analysis of variance
