# Supplementary material for: The Effects of Transcranial Direct Current Stimulation on Dual-Task Interference Depend on the Dual-Task Content
Source: Front Hum Neurosci. 2021 Mar 26;15:653713. doi: 10.3389/fnhum.2021.653713 (PMC8032873; doi:10.3389/fnhum.2021.653713)
Supplement: Supplementary file 3 [file Table_3.DOCX]

**Supplemental table S3.** Result of the three-way repeated-measures ANOVA on variable in the Stroop task under the single- and dual-task condition

| **Single-task condition** | Stroop task | | | |
| --- | --- | --- | --- | --- |
|  | F value | p value | partial η^2^ | 1-β |
| Placement | 0.476 | 0.508 | 0.050 | 0.429 |
| Polarity | 0.115 | 0.742 | 0.013 | 0.146 |
| Time | 0.828 | 0.490 | 0.084 | 0.679 |
| Placement × Polarity | 7.363 | 0.024 | 0.450 | 1.000 |
| Placement × Time | 0.119 | 0.948 | 0.013 | 0.191 |
| Polarity × Time | 0.309 | 0.819 | 0.033 | 0.412 |
| Placement × Polarity × Time | 1.246 | 0.313 | 0.122 | 0.641 |
|  |  | |  |  |
| **Dual-task condition** | Stroop-tandem dual-task | | | |
|  | F value | p value | partial η^2^ | 1-β |
| Placement | 1.799 | 0.213 | 0.167 | 0.932 |
| Polarity | 0.381 | 0.553 | 0.041 | 0.363 |
| Time | 0.226 | 0.878 | 0.024 | 0.242 |
| Placement × Polarity | 4.213 | 0.070 | 0.319 | 1.000 |
| Placement × Time | 0.569 | 0.640 | 0.060 | 0.661 |
| Polarity × Time | 0.216 | 0.885 | 0.023 | 0.304 |
| Placement × Polarity × Time | 1.974 | 0.142 | 0.180 | 0.830 |

Abbreviations: ANOVA, analysis of variance
