# Supplementary material for: The Effects of Transcranial Direct Current Stimulation on Dual-Task Interference Depend on the Dual-Task Content
Source: Front Hum Neurosci. 2021 Mar 26;15:653713. doi: 10.3389/fnhum.2021.653713 (PMC8032873; doi:10.3389/fnhum.2021.653713)
Supplement: Supplementary file 4 [file Table_4.DOCX]

**Supplemental table S4.** Result of combined analysis in the tandem task (the result of the four-way repeated-measures ANOVA on variable in the tandem task)

|  | Single-task and word-tandem dual-task | | | |  | Single-task and Stroop-tandem dual-task | | | |
| --- | --- | --- | --- | --- | --- | --- | --- | --- | --- |
|  | F value | p value | partial η^2^ | 1-β |  | F value | p value | partial η^2^ | 1-β |
| Condition | 15.234 | 0.004 | 0.629 | 1.000 |  | 15.930 | 0.003 | 0.639 | 1.000 |
| Placement | 2.293 | 0.164 | 0.203 | 0.999 |  | 2.543 | 0.145 | 0.220 | 0.999 |
| Polarity | 0.482 | 0.505 | 0.051 | 0.664 |  | 0.472 | 0.509 | 0.050 | 0.655 |
| Time | 0.933 | 0.395 | 0.094 | 0.944 |  | 0.844 | 0.430 | 0.086 | 0.922 |
| Condition × Placement | 0.718 | 0.419 | 0.074 | 0.913 |  | 0.203 | 0.663 | 0.022 | 0.426 |
| Condition × Polarity | 3.576 | 0.091 | 0.284 | 1.000 |  | 1.449 | 0.259 | 0.139 | 0.997 |
| Condition × Time | 0.630 | 0.602 | 0.065 | 0.914 |  | 1.013 | 0.402 | 0.101 | 0.988 |
| Placement × Polarity | 6.802 | 0.028 | 0.430 | 1.000 |  | 3.403 | 0.098 | 0.274 | 1.000 |
| Placement × Time | 0.009 | 0.999 | 0.001 | 0.068 |  | 0.452 | 0.718 | 0.048 | 0.809 |
| Polarity × Time | 0.183 | 0.907 | 0.020 | 0.439 |  | 0.509 | 0.680 | 0.053 | 0.847 |
| Condition × Placement × Polarity | 1.102 | 0.321 | 0.109 | 0.999 |  | 4.725 | 0.058 | 0.344 | 1.000 |
| Condition × Placement × Time | 0.115 | 0.950 | 0.013 | 0.424 |  | 0.636 | 0.598 | 0.066 | 0.983 |
| Condition × Polarity × Time | 1.570 | 0.219 | 0.149 | 1.000 |  | 1.812 | 0.169 | 0.168 | 1.000 |
| Placement × Polarity × Time | 3.694 | 0.024 | 0.291 | 1.000 |  | 1.994 | 0.139 | 0.181 | 1.000 |
| Condition × Placement × Polarity × Time | 1.437 | 0.254 | 0.138 | 1.000 |  | 0.976 | 0.418 | 0.098 | 1.000 |

Abbreviations: ANOVA, analysis of variance
