# Supplementary material for: The Effects of Transcranial Direct Current Stimulation on Dual-Task Interference Depend on the Dual-Task Content
Source: Front Hum Neurosci. 2021 Mar 26;15:653713. doi: 10.3389/fnhum.2021.653713 (PMC8032873; doi:10.3389/fnhum.2021.653713)
Supplement: Supplementary file 5 [file Table_5.DOCX]

**Supplemental table S5.** Result of combined analysis in the word task (the result of the four-way repeated-measures ANOVA on variable in the word task)

|  | Single-task and word-tandem dual-task | | | |
| --- | --- | --- | --- | --- |
|  | F value | p value | partial η^2^ | 1-β |
| Condition | 0.530 | 0.485 | 0.056 | 0.706 |
| Placement | 0.863 | 0.377 | 0.087 | 0.885 |
| Polarity | 1.069 | 0.328 | 0.106 | 0.941 |
| Time | 4.793 | 0.008 | 0.348 | 1.000 |
| Condition × Placement | 0.001 | 0.977 | <0.001 | <0.001 |
| Condition × Polarity | 0.047 | 0.834 | 0.005 | 0.134 |
| Condition × Time | 0.476 | 0.702 | 0.050 | 0.826 |
| Placement × Polarity | 2.163 | 0.175 | 0.194 | 1.000 |
| Placement × Time | 2.812 | 0.058 | 0.238 | 1.000 |
| Polarity × Time | 1.950 | 0.145 | 0.178 | 1.000 |
| Condition × Placement × Polarity | 0.882 | 0.372 | 0.089 | 0.994 |
| Condition × Placement × Time | 0.389 | 0.762 | 0.041 | 0.889 |
| Condition × Polarity × Time | 0.100 | 0.959 | 0.011 | 0.368 |
| Placement × Polarity × Time | 1.662 | 0.199 | 0.156 | 1.000 |
| Condition × Placement × Polarity × Time | 2.381 | 0.092 | 0.209 | 1.000 |

Abbreviations: ANOVA, analysis of variance
