# Supplementary material for: The Effects of Transcranial Direct Current Stimulation on Dual-Task Interference Depend on the Dual-Task Content
Source: Front Hum Neurosci. 2021 Mar 26;15:653713. doi: 10.3389/fnhum.2021.653713 (PMC8032873; doi:10.3389/fnhum.2021.653713)
Supplement: Supplementary file 6 [file Table_6.DOCX]

**Supplemental table S6.** Result of combined analysis in the Stroop task (the result of the four-way repeated-measures ANOVA on variable in the Stroop task)

|  | Single-task and Stroop-tandem dual-task | | | |
| --- | --- | --- | --- | --- |
|  | F value | p value | partial η^2^ | 1-β |
| Condition | 0.173 | 0.687 | 0.019 | 0.300 |
| Placement | 1.099 | 0.322 | 0.109 | 0.947 |
| Polarity | 0.220 | 0.650 | 0.024 | 0.367 |
| Time | 0.295 | 0.829 | 0.032 | 0.520 |
| Condition × Placement | 6.749 | 0.029 | 0.429 | 1.000 |
| Condition × Polarity | 0.173 | 0.687 | 0.019 | 0.376 |
| Condition × Time | 2.426 | 0.087 | 0.212 | 1.000 |
| Placement × Polarity | 5.779 | 0.040 | 0.391 | 1.000 |
| Placement × Time | 0.379 | 0.769 | 0.040 | 0.731 |
| Polarity × Time | 0.446 | 0.722 | 0.047 | 0.800 |
| Condition × Placement × Polarity | 2.909 | 0.122 | 0.244 | 1.000 |
| Condition × Placement × Time | 0.291 | 0.832 | 0.031 | 0.786 |
| Condition × Polarity × Time | 0.026 | 0.994 | 0.003 | 0.135 |
| Placement × Polarity × Time | 1.484 | 0.241 | 0.142 | 1.000 |
| Condition × Placement × Polarity × Time | 1.746 | 0.181 | 0.162 | 1.000 |

Abbreviations: ANOVA, analysis of variance
